# The impact of Heads Up testing on thrombectomy for acute ischemic stroke

**DOI:** 10.3389/fstro.2025.1612019

**Published:** 2025-07-21

**Authors:** Mallory Blackwood, Charles Beaman, Latisha Sharma, David S. Liebeskind

**Affiliations:** University of California Los Angeles (UCLA) Department of Neurology, Los Angeles, CA, United States

**Keywords:** ischemic stroke (acute), mechanical thrombectomy, low NIHSS, collateral perfusion, large vessel occlusion

## Abstract

The Heads Up test, initially described in 2017, offers a potential tool for assessing likelihood of collateral failure in patients with acute large vessel occlusion (LVO) but low or resolving NIH who may become candidates for mechanical thrombectomy (MT). By raising the head of bed and performing serial exams (Heads Up test), detection of early symptomatic worsening may indicate vulnerability of collateral blood supply. The present study aims to examine the practical applications and outcomes of the Heads Up test in one institution over 9 years by analyzing 15 consecutive cases of documented Heads Up testing. Our findings suggest that the Heads Up test can provide valuable guidance in treatment decisions, but further data is needed to refine its criteria and applicability in the evolving neurointerventional practice.

## Introduction

Since its inception, mechanical thrombectomy (MT) has revolutionized acute ischemic stroke treatment, especially for acute large vessel occlusion (LVO) strokes (Lambrinos et al., 2016). The Heads Up test, described in the literature in 2017, was developed to support decision-making for MT in cases where patients present with LVO but exhibit only mild neurological symptoms after being supine during initial assessment (Ali et al., 2016). By elevating the head-of-bed to 90 degrees for a duration of 30 min and assessing for change in the NIH Stroke Scale (NIHSS), the Heads Up test aims to identify patients at risk of functional deterioration. In theory, any worsening upon assuming the upright position may indicate impending collateral vulnerability warranting intervention. Therefore, a positive Heads Up test is defined as any worsening of neurological status during the test period, prompting its termination and immediate catheterization of the patient.

At the time of the cases described by Ali et al., criteria for MT were poorly defined and have since been cemented and expanded in several landmark trials (Berkhemer et al., 2015; Campbell et al., 2015; Saver et al., 2015). By examining the use of this test in the years following these landmark trials, we may have a better sense of its usefulness in the modern era of MT. The landscape of neurointerventional has further evolved in the setting of more advanced imaging techniques (Jadhav et al., 2021; Pfaff et al., 2016). Increasingly, perfusion imaging is used to determine eligibility for MT, especially in the setting of minor or resolving neurological deficit. Based on its proposed mechanism, Heads Up testing may be thought of as a clinical test of related perfusion-dependent pathology. Here, we present a retrospective analysis of real-world Heads Up test use at our institution between 2015 and 2024, discussing the patient demographics, procedural outcomes, and complications in 15 cases to further elucidate the clinical role of Heads Up testing in determining MT candidacy in this evolving clinical environment.

## Methods

This is a monocentric retrospective review which analyzed 15 patients with acute LVO stroke who underwent Heads Up testing at our institution between 2015 and 2024. Charts were reviewed to acquire patient demographics, NIHSS scores, Heads Up testing rationale, test outcomes, and treatment decisions. Patient comorbidities, prior antiplatelet or anticoagulation use, and details of intervention were also reviewed.

The Heads Up test was performed by elevating the patient's head to 90 degrees and monitoring for any NIHSS changes over 30 min. The test was considered positive if any deterioration occurred, prompting catheterization and consideration for MT. The decision to utilize Heads Up was made by the stroke and interventional teams, typically in the context of a low or resolving NIH with the potential for disabling deficit. Because of this, no presenting NIHSS cutoffs were used in case selection.

Cases were identified from a database of acute strokes at our institution and then refined by a search for “Heads Up” mentioned in documentation. This was followed by confirmation with individual chart review, yielding only cases in which Heads Up was documented correctly and performed in the IR suite. Interventions, including MT techniques (e.g., aspiration, stent retrievers) and adjunctive procedures (e.g., angioplasty, stenting), were recorded. Patient outcomes, including expanded Thrombolysis in Cerebral Infaction (eTICI) scale, symptomatic intracranial hemorrhage (ICH, defined as any clinical worsening with Parenchymatous Hematoma 1 or greater bleed per the Heidelberg criteria) and NIHSS scores post-intervention, were analyzed (Liebeskind et al., 2019; von Kummer et al., 2015).

A Negative Predictive Value was then calculated based on a formula of NPV = 100 ^*^ [True Negative/(True Negative +False Negative)] wherein true negative represents Heads Up-Negative cases where MT was never performed, and false negative represents Heads Up-Negative cases where MT was performed during hospitalization. A Positive Predictive Value was calculated based on a formula or PPV = 100 ^*^ [True Positive/(True Positive + False Positive)] wherein true positive represented Heads Up-Positive cases who received MT during hospitalization and false positive represented Heads Up-Positive cases where MT was not performed.

## Results

Identifying Heads Up cases proved difficult due to a lack of standardized documentation. Cases were identified by a search of the Electronic Health Record (EHR), and only cases where Heads Up testing in the Interventional Radiology (IR) suite was mentioned, with documented exam, were included in the assessment.

Of the 15 patients who underwent Heads Up testing, four demonstrated worsening neurological function and were classified as Heads Up-positive. Eleven patients did not demonstrate neurological worsening and were classified as Heads Up-negative. In Four patients, thrombectomy was attempted immediately following Heads Up testing, though of note, one of these was performed despite a negative Heads Up result, based on several patient factors and a risk/benefit discussion. In addition, one patient with positive heads up received thrombolysis and later angiography, but a thrombectomy was not performed due to the location of the clot. Ultimately, MT was performed in seven cases during the hospital stay; four cases were conducted immediately following Heads Up testing, and three cases required delayed intervention due to clinical worsening. All seven MT cases achieved eTICI 2b or higher, indicating successful revascularization. The negative predictive value of the Heads Up test in our series was therefore 63%. The positive predictive value of the Heads Up test in our series was therefore 75%.

A combined approach of aspiration and stent retrievers was most common, with six of eight cases achieving favorable outcomes, while aspiration alone was effective in only two cases. Two patients required permanent stent placement, and one required angioplasty for significant intracranial stenosis. Symptomatic ICH occurred in two cases: one who received MT and one who did not, both of whom were initially Heads Up-negative.

Fourteen of the 15 (93%) patients had anterior circulation occlusion, three (20%) had stenosis of the affected vessel. Patient demographics (summarized in Table 1) revealed a high prevalence of cardiovascular risk factors: in the entire cohort, 53% had hypertension, 40% had hyperlipidemia, and 13.3% had malignancy or hypercoagulability. Notably, eight patients (53.3%) were on antiplatelet or anticoagulant therapy before admission. Other comorbidities included coronary artery disease (20%), atrial fibrillation (20%), prior stroke (6.6%), and diabetes (6.6%). These demographics are further reported on the basis of the two groups (Head Up-positive and Heads-Up Negative) in Table 1.

**Table 1:** Demographics by test outcome.

**Demographics**	**Heads Up positive (*N* = 4)**	**Heads Up negative (*N* = 11)**
Mean age (±SD)	61(±29)	66(±19)
Gender # male (%)	1 (25%)	4 (36%)
Occlusion location (%M1/M2)	75%	72%
Mean initial NIHSS (±SD)	3.5 (±2.5)	4.3 (±6.3)
Average time to presentation (min)	217 (±299)	359 (±345)
Previous antiplatelet/anticoagulation	2 (50%)	6 (54%)
Prior hypertension	2 (50%)	6 (54%)
Prior hyperlipidemia	0 (0%)	6 (54%)
Prior malignancy/hypercoagulability	1 (25%)	1 (9%)
Prior stroke	1 (25%)	0 (0%)
Suspected ICAD	2 (50%)	5 (45%)
Prior atrial fibrillation	0 (0%)	3 (27%)
Prior diabetes	1 (25%)	0 (0%)

**Figure 1:**
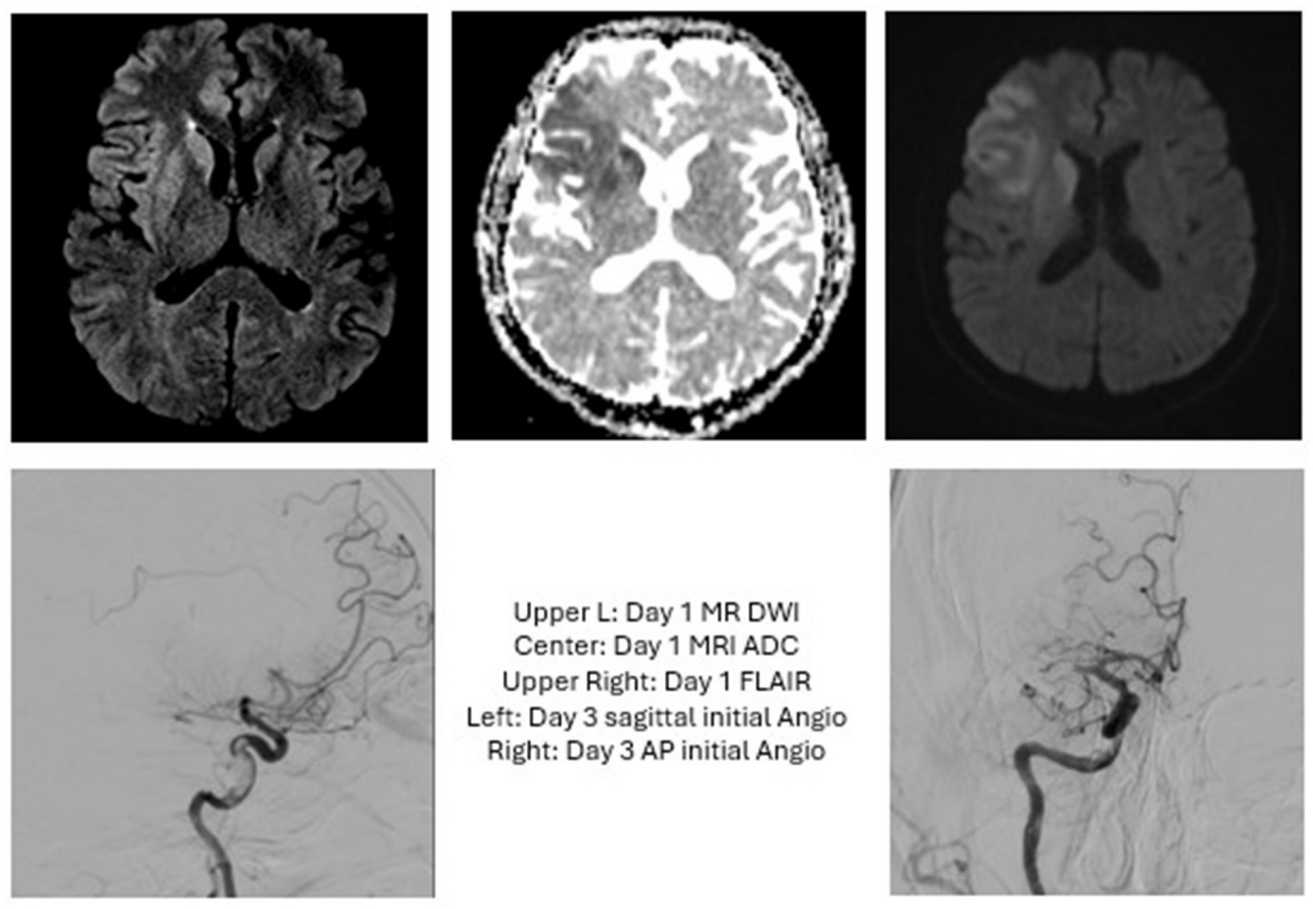
Case 1: The patient presented with an initial NIHSS of 7 and a R ICA occlusion but improved following administration of tPA. Heads Up testing in the IR suite revealed no further worsening, so MT was deferred. Three days later, the patient acutely worsened with NIHSS of 10 and an MRI showing R MCA occlusion. MT was performed with TICI 2C. Post-proceduraly, this patient required decompressive hemicraniectomy and was discharged with a tracheostomy and G-tube.

**Figure 2:**
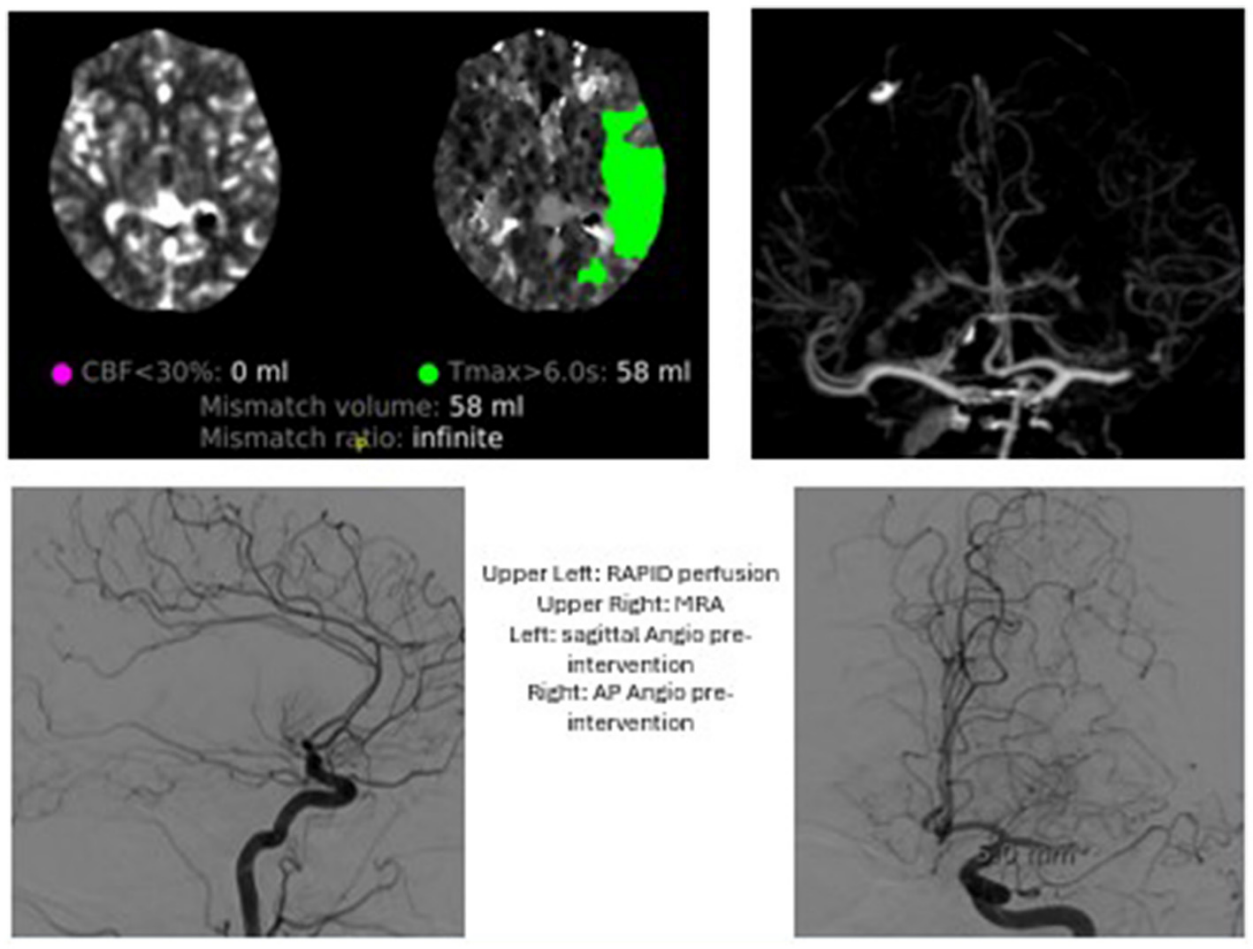
Case 2: The patient presented with wake up stroke and an NIHSS of 6. An MRA at that time revelaed a L M1 occlusion. A Heads Up test in IR suite was positive and MT performed with TICI2C. The patient was discharged with minimal new deficit.

**Figure 3:**
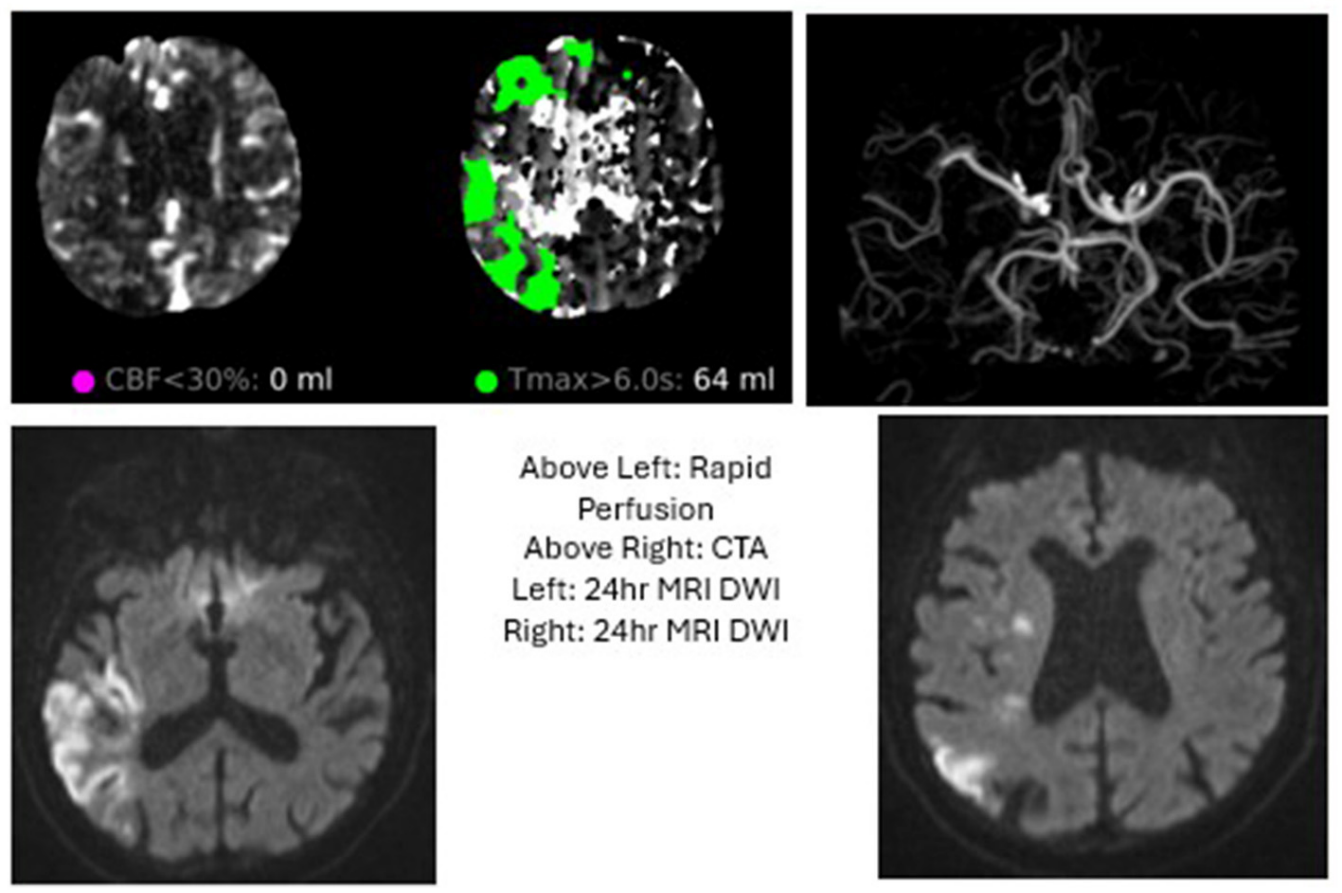
Case 3: The patient was found down with a R MCA syndrome and NIHSS of 6 with a cutoff at R M2, but demonstrated no change in exam upon Heads Up testing. The patient was admitted to ICU and had slow improvement in his exam over subsequent days.

Three illustrative cases, along with relevant imaging, are described in Figures 1–3. These were selected as typical examples of the three possible testing outcomes: initial Heads Up-Negative with need for later intervention (Figure 1), initial Heads Up-positive with intervention performed immediately (Figure 2) and initial Heads Up-negative without need for later intervention (Figure 3).

## Discussion

Our cohort offers insights into the clinical utility of Heads Up testing for assessing MT candidacy in patients with acute LVO stroke and low NIHSS scores. MT inclusion criteria have expanded in recent years, and yet, invasive catheterization itself comes with inherent risk. Therefore, it is important to refine our clinical assessment tools alongside burgeoning imaging techniques. The Heads Up test may be one such clinical tool. At our institution, Heads Up testing was most frequently employed in cases where there was uncertainty regarding the need for intervention, such as minor or resolving neurological deficit, complex medical history or imaging suggestive of proximal occlusion despite minimally disabling symptoms.

Our findings suggest that approximately half of Heads Up-tested patients required MT during hospitalization, with three cases initially deemed Heads Up-negative later requiring intervention due to neurological worsening. This relatively high proportion suggests that the very impulse to utilize Heads Up testing may predict some impending collateral failure. The reasons for these cases testing negative remain unclear, but it may be the case that 30 min is an insufficient length of time or that other risk factors (hypercoagulability, prior stroke) may confer further vulnerability. Overall, the Heads Up test in our small series demonstrated a positive predictive value of 75% indicating that it may be helpful to rule out the need for MT in certain patients. This highlights the Heads Up test's potential, and even the intuition to utilize it, though further studies are needed to refine criteria for Heads Up-positive status and to investigate potentially lengthening the testing period.

The need for adjunctive procedures (e.g., stenting, angioplasty) in several cases indicates that patients with Heads Up-positive tests may have more complex vascular pathology, potentially predisposing them to re-occlusion. Moreover, the observed rate of symptomatic ICH underscores the importance of cautious patient selection, particularly in those with existing comorbidities, anticoagulation therapy, or delays in decision to intervene.

This study emphasizes the evolving criteria for MT and suggests that Heads Up testing may offer a valuable decision-making tool, especially as inclusion criteria for MT expand. An evolving NIHSS is critical to mechanical thrombectomy decision. To do this, positional changes may need to be evaluated with a standard protocol as we have defined with Heads Up. To allow for better quantification of the decision to pursue mechanical thrombectomy in real practice, we recommend that interventionalists familiarize themselves with the Heads Up test and document it in their exam when applicable. Given the limited sample size and observational nature of this study, further research is warranted to validate Heads Up testing as a reliable predictor of MT benefit. Future studies should aim to establish standardized protocols for Heads Up testing and evaluate its predictive value in a diverse patient population.

## Limitations

While some conclusions may be drawn from this retrospective case series, there are of course limitations. The sample size is smaller than expected, in large part due to insufficient documentation. In addition, the retrospective and monocentric nature carries its own limitations, as the decision to perform Heads Up testing was made by the treatment team without a standardized criteria for when it should be applied. We can also not assess the “false positives” or patients who underwent thrombectomy that may not have required it were they subjected to a Heads Up test.

## Conclusion

In this small cohort, Heads Up testing served as a useful adjunct in the decision-making process for MT in acute LVO patients with low or resolving NIHSS. Approximately half of Heads Up-tested patients ultimately required MT, with successful outcomes in cases achieving TICI 2b or higher. Given this, the impulse to utilize Heads Up may in itself predict eventual decompensation. The decision to pursue MT in acute stroke, especially those with low/resolving NIH is a nebulous one which will ultimately depend upon patient and physician factors such as how debilitating the symptoms are, location of thrombus, and pre-morbid state. The Heads Up test shows promise for identifying patients at risk of deterioration and potential benefit from MT; however, larger studies are necessary to optimize its role in neurointerventional protocols. Further research should focus on refining Heads Up test criteria and integrating it with evolving perfusion imaging grading to enhance patient outcomes in acute ischemic stroke.

## Data Availability

The raw data supporting the conclusions of this article will be made available by the authors, without undue reservation.

## References

[B1] Ali L. K. Weng J. K. Starkman S. Saver J. L. Kim D. Ovbiagele B.. (2016). Heads up! A novel provocative maneuver to guide acute ischemic stroke management. Interven. Neurol. 6:8. 10.1159/000449322 28611828 PMC5465685

[B2] Berkhemer O. A. Fransen P. S. S. Beumer D. Berg L. A. van den Lingsma H. F. Yoo A. J.. (2015). A randomized trial of intraarterial treatment for acute ischemic stroke. N. Engl. J. Med. 372, 11–20. 10.1056/NEJMoa1411587 25517348

[B3] Campbell B. C. V. Mitchell P. J. Kleinig T. J. Dewey H. M. Churilov L. Yassi N.. (2015). Endovascular therapy for ischemic stroke with perfusion-imaging selection. N. Engl. J. Med. 372, 1009–1018. 10.1056/NEJMoa1414792 25671797

[B4] Jadhav A. P. Desai S. M. Jovin T. G. (2021). Indications for mechanical thrombectomy for acute ischemic stroke: current guidelines and beyond. Neurology 97(20 Suppl. 2), S126–S136. 10.1212/WNL.0000000000012801 34785611

[B5] Lambrinos A. Schaink A. K. Dhalla I. Krings T. Casaubon L. K. Sikich N.. (2016). Mechanical thrombectomy in acute ischemic stroke: a systematic review. Can. J. Neurol. Sci. 43, 455–460. 10.1017/cjn.2016.30 27071728 PMC4926268

[B6] Liebeskind D. S. Bracard S. Guillemin F. Jahan R. Jovin T. G. Majoie C. B.. (2019). eTICI reperfusion: defining success in endovascular stroke therapy. J. Neurointerven. Surg. 11, 433–438. 10.1136/neurintsurg-2018-014127 30194109

[B7] Pfaff J. Herweh C. Pham M. Schönenberger S. Nagel S. Ringleb P. A.. (2016). Mechanical thrombectomy in patients with acute ischemic stroke and lower NIHSS scores: recanalization rates, periprocedural complications, and clinical outcome. Am. J. Neuroradiol. 37, 2066–2071. 10.3174/ajnr.A4862 27365324 PMC7963792

[B8] Saver J. L. Goyal M. Bonafe A. Diener H.-C. Levy E. I. Pereira V. M.. (2015). Stent-retriever thrombectomy after intravenous t-PA vs. T-PA alone in stroke. N. Engl. J. Med. 372, 2285–2295. 10.1056/NEJMoa1415061 25882376

[B9] von Kummer R. Broderick J. P. Campbell B. C. V. Demchuk A. Goyal M. Hill M. D.. (2015). The Heidelberg bleeding classification. Stroke 46, 2981–2986. 10.1161/STROKEAHA.115.010049 26330447

